# From Non-Traditional Sexual Behavior to Non-Legitimate Victims: Moral Virtue, Victim Blame, and Helping Intentions Toward a Woman Victim of Image-Based Sexual Abuse

**DOI:** 10.1007/s10508-024-02970-x

**Published:** 2024-09-05

**Authors:** Maria Giuseppina Pacilli, Stefano Pagliaro, Ilaria Giovannelli, Federica Spaccatini, Elisa Berlin, Chiara Rollero

**Affiliations:** 1 Department of Political Sciences, University of Perugia, Via Elce Di Sotto, 06123 Perugia, Italy; 2 Department of Psychology, University of Chieti-Pescara, Chieti, Italy; 3 Department of Psychology, University of Turin, Turin, Italy

**Keywords:** Morality, Victim blame, Image-based sexual abuse, Sexting, Gender-based violence

## Abstract

Image-based sexual abuse represents an increasingly common form of gender-based violence, consisting of the act of non-consensually capturing, distributing, or threatening to distribute sexually explicit material depicting another person. The purpose of the present study was to investigate how women victims’ noncompliance with traditional female sexuality influences bystanders’ perceptions of the phenomenon. Specifically, we experimentally examined whether a woman’s sexual agency (high vs. low) and the length (steady vs. transient) of the relationship with the perpetrator affected her moral evaluation, victim blaming, and participants’ willingness to support her. A sample of 597 adults (65.7% women, *M*_*age*_ = 31.29 years) took part in the study. The findings indicated that while a transient (vs. steady) relationship with the perpetrator significantly lowered the woman’s perceived moral virtue and increased the extent to which she was blamed for the incident, a high (vs. low) woman’s sexual agency decreased participants’ helping intentions towards her. Additionally, results showed that men were less likely than women to attribute moral virtue and help the victim. Lastly, through the mediation of moral virtue and victim blaming, the length of the relationship indirectly influenced participants’ helping intentions.

## Introduction

Image-based sexual abuse is a severe form of sexual violence that relies on technology to non-consensually capture, distribute, or threaten to distribute nude or sexual images of another person (Powell et al., 2019). Recent studies show that this phenomenon is becoming more common (Patel & Roesch, 2022; Powell et al., 2022) and that women are victimized more than men in several Western countries (Gove, 2022; Permessonegato, 2022; Ruvalcaba & Eaton, 2019).

In patriarchal cultures, women have often been stereotyped as the “guardians of sexuality,” which places a disproportionate amount of responsibility for sexual conduct on them (Abrams et al., 2003). Despite the enduring influence of cultural norms regarding women’s presumed legitimate sexuality (Elakkary et al., 2014; Pacilli et al., 2022), there is a lack of research investigating how deviating from traditional female sexuality may affect attitudes towards a woman who has been a victim of image-based sexual abuse. To address this gap, in the present experimental study, we aimed to investigate how bystanders perceive a woman based on her non-compliance with traditional female sexuality in terms of sexual agency and the length of her relationship with the man with whom she recorded sexual intercourse.

When individuals who are victims of violence violate stereotypical norms through their actions, they distance themselves from the ideal image of a legitimate victim who is worthy of support and are more likely to be blamed for what has happened (Carpenter, 2005). In Italy, an emblematic case of negative reactions towards a perceived non-legitimate victim of image-based sexual abuse is that of Tiziana Cantone, who tragically committed suicide in 2016. The distribution of six sexually explicit videos featuring Cantone was non-consensual, and in one of the videos, she could be heard asking the person holding the camera, “Are you filming? Bravo!”. The woman’s behavior in the video clearly indicated a deviation from conventional norms of female sexual modesty and self-restraint. Specifically, in a non-dyadic and stable relationship, she exhibited clear sexual agency by explicitly asking to be filmed during sexual activity and expressing pleasure at that (Reynolds, 2017). Regrettably, this sequence rapidly circulated on prominent porn websites, was incorporated into satirical audiovisual products, and powerfully contributed to her public humiliation and condemnation (Romania, 2022).

### Perception of Women Who Deviate from Traditional Sexuality

In contemporary Western society, female sexuality is still subject to heightened scrutiny compared to male sexuality, often enforced through the mechanism of sexual reputation (Farvid et al., 2017). In order to protect their reputation, women are expected to adhere to normative expectations of submissiveness and exhibit sexual reactivity and passivity differently from men, who are expected to exhibit sexual proactivity, dominance, and initiation in heterosexual encounters (Champion et al., 2022; Zaikman et al., 2016).

While there have been some changes in societal expectations surrounding the conception of female sexuality, women still face constraints on their ability to exercise autonomy over their sexual lives (Zvi, 2022). For instance, there is still widespread bias against women who engage in casual sex, which is perceived as disadvantageous for women as well as indicating underlying psychological issues (Crawford & Popp, 2003). Conley et al. (2013) found that when it comes to accepting casual sex offers, women are subject to more negative perceptions than men. Specifically, women who agreed to such offers were judged to be more promiscuous, less mentally healthy, less intelligent, and riskier compared to men who accepted similar offers. Accordingly, through six experiments, Krems et al. (2021) showed that women who engage in casual sex are negatively stereotyped by both genders, while men are not subjected to the same stereotype.

To our knowledge, there is a lack of research exploring how deviation from traditional female sexuality may affect attitudes toward a woman who has been a victim of image-based sexual abuse. Relevant insights arise from studies examining the effects of sexualized clothing practices (which could allude to sexual freedom and disinhibition) on social perception. Research has shown that sexualized appearance negatively impacts the perception of victims of extreme and blatant gender violence, with sexualized victims being blamed more, and seen as less traumatized, and needing less time to recover (Loughnan et al., 2013; Pacilli et al., 2017; Spaccatini et al., 2019, 2023). Examining perception of a female victim who had taken intimate images of herself for personal use–but had no awareness of how they were disseminated on the internet–Zvi (2022) showed how she faced greater victim blaming compared to the case where the images were covertly taken by an ex-intimate partner.

### Bystanders’ Perception of Gender Violence Victims

Bystanders are individuals who are not directly involved in an emergency situation but, by their mere presence, possess the potential to either do nothing, exacerbate the problem by condoning the perpetrator’s behavior, or be unsupportive toward the victim, as well as intervene and stop a high-risk situation by offering assistance and improving the situation (Banyard, 2011). Since the classical model by Latané and Darley (1970), research has shed light on the crucial role of bystanders as a source of prevention and support in emergency situations, considering that multiple barriers to intervention are present (for a review, see Burn, 2009). For instance, bystanders may fail to notice the situation because they do not recognize it as dangerous. Thus, investigating factors that might increase the likelihood of a bystander interpreting the situation unequivocally as an emergency and accepting responsibility for intervention is pivotal.

Recently, applying the bystander approach to the examination of intimate partner violence, a solid line of research (Gramazio et al., 2021; Pacilli et al., 2017, 2022; Pagliaro et al., 2020, 2021a, b) has shown how the decision to help a victim is mediated by various factors that contribute to perceiving that target as worthy of assistance. As regards bystanders’ characteristics, gender has been examined, showing that women are more willing to intervene than men (Bryant & Spencer, 2003; Pacilli et al., 2017; Spaccatini et al., 2023, but see Banyard et al., 2020). Moreover, it has been shown that morality plays a crucial role in influencing bystanders’ reaction. Moral judgments are likely to prompt internal attribution more strongly than other types of judgments (e.g., competence or sociability) because individuals tend to perceive morality-based evaluations to be more stable over time and reflect the true nature of a person (Pagliaro et al., 2016). For instance, when a victim admitted infidelity or exhibited sexualized appearances (Gramazio et al., 2021; Pacilli et al., 2017), bystanders evaluated her as less moral. The (im)morality attributed to the victim resulted in increased victim blaming (Ellison & Munro, 2009; Pacilli et al., 2022; Pagliaro et al., 2016; Penone & Spaccatini, 2019). This, in turn, diminished the intention to help and support the victim (Baldry et al., 2015; Pacilli et al., 2017, 2022; Pagliaro et al., 2020, 2021a).

If the role of bystanders is fundamental in addressing conventional forms of violence, it becomes even more critical in the context of non-consensual dissemination of sexual images, where the number of bystanders capable of either preventing or exacerbating the dissemination is potentially infinite. While research has increasingly scrutinized victim blaming and slut shaming directed toward women who engage in sexting and, more broadly, victims of image-based sexual abuse (Flynn et al., 2023; Maes et al., 2023; Naezer & van Oosterhout, 2021), still less research is available on the potential crucial role of the moral perception of the victims of image-based sexual abuse and how it affects victim blaming and helping intentions. The focus on the perceived morality of the victim is particularly relevant within this context of violence, given the strong association between women’s morality and their perceived legitimate sexuality (Elakkary et al., 2014), with moral values such as chastity and purity historically linked to female sexual abstinence and restraint (Melandri, 2018). Therefore, it is crucial to understand whether and how victims’ morality affects victim blaming and the extent of bystanders’ helping intentions based on the victim’s sexual behavior.

### The Present Research

The aim of the present research was to investigate whether bystanders’ perceptions of a woman victim of image-based sexual abuse varied according to the degree to which she deviated from traditional female sexuality in terms of sexual agency and the length of her relationship with the man with whom she recorded the sexual intercourse. We experimentally examined whether a woman’s sexual agency (high vs. low) and the length (steady vs. transient) of the relationship with the perpetrator affected her moral evaluation, victim blaming, and willingness to support the victim.

The extant academic literature pertaining to the non-consensual dissemination of intimate images indicates that heterosexual men frequently employ coercive tactics to elicit such images from their female counterparts (Burkett, 2015). While instances of men producing sexual imagery of women without their consent are not uncommon, women may also actively choose to engage in such behavior with a male partner. However, to the best of our knowledge, no empirical research has investigated individuals’ attitudes toward victims based on their level of sexual agency in the context of sex video creation. Conversely, the few studies exploring the impact of relationship duration with the perpetrator on victims’ perspectives of the non-consensual dissemination of intimate material have produced contradictory findings. While Bothamley and Tully (2018) demonstrated that victims were less likely to be blamed when in a one-year relationship compared to a one-month relationship, Starr and Lavis (2018) found no significant effect of perpetrator-victim relationship length on victim blame.

However, it is plausible that the victim’s evaluation may vary depending on the circumstances surrounding the sexual activity. A woman who fits (vs. does not fit) the traditional female sexuality because she was coerced into filming herself may be perceived as less culpable: she is participating in a “less illegitimate” sexual activity because of coercion and, therefore, she is passively complying (as women are expected to do) with someone else’s desires. Consistently, a woman who engages in such behavior with a long-term partner out of affection and within the context of a conventional relationship may be perceived as less transgressing the boundaries of female traditional sexuality, which emphasizes the importance of engaging in sexuality only within romantic relationships. As a result, she may elicit less disapproval from bystanders.

Based on the previous considerations and aforementioned research literature, we hypothesized that women who proactively (vs. not proactively) take sexual images of themselves during sexual intercourse (Hp1a), as well as those who do so in the context of a transient (vs. steady) relationship (Hp1b), may be perceived as more immoral because they deviate from the expected traditional sexual passivity and restraint. We explored gender differences in moral evaluation as well as the interaction among our other independent variables. In addition, we anticipated that women who proactively (vs. not proactively) take sexual images of themselves or who do so in the context of a transient (vs. steady) relationship may be perceived as more responsible for the violence perpetrated against them (respectively, Hp2a and Hp2b) and less deserving of help (respectively, Hp3a and Hp3b). As for the gender of the perceivers, the inconsistent study literature prevents definite hypotheses; thus, we investigate whether there are gender differences in victim blaming. In terms of helping intent, based on the literature, we anticipate that women may be more likely than men to assist the victim (Hp3c). Finally, we hypothesized that the relationship between the victims’ sexual agency (Hp4a) and the relationship length (Hp4b) and perceivers’ willingness to help her may be sequentially mediated by moral virtue and victim blaming. Previous research has primarily examined attitudes toward victims of non-consensual dissemination of sexual images without considering the circumstances surrounding the sexual activity (e.g., Maes et al., 2023; Rollero & Pagliaro, 2022).

## Method

### Design and Participants

We conducted a 2 × 2 (Sexual Agency: Low vs. High) × (Relationship’s Length: Steady vs. Transient) between participants factorial experimental design.

An a priori power analysis was conducted for sample size estimation (using GPower 3.1.; Faul et al., 2007; Schoemann et al., 2017). With an alpha = 0.05 and power = 0.80, the projected sample size needed to detect a medium effect size (*f *= 0.25) was at least *N* = 128 for a between-groups comparison (one-way ANCOVA with four groups and a covariate; main effects and interactions). As for mediation analysis, based on smallest correlations among mediators and dependent variable available in research literature (Pagliaro et al., 2021b), and assuming, at worst, weak correlations among the independent variable and the other variables (*r* = 0.15) we estimated that with power = 0.80 and 10,000 resample, we needed a sample composed of at least 370 participants (Schoemann et al., 2017). We recruited 600 individuals from the general population and randomly assigned them to one of four experimental conditions. Specifically, trained students attending the last year of the Faculty of Psychology adopted snowball sampling techniques to recruit participants from the general Italian population among their interpersonal networks. The survey was spread via web platforms and social networks. After excluding three participants who provided contradictory and unreliable information (e.g., age of 99 years conflicting with educational background), the retained sample consisted of 597 individuals. Among the retained participants, nine of them failed to accurately recall the experimental manipulation; however, the inclusion or exclusion of these individuals did not affect the results. Therefore, the final sample comprised 597 individuals (65.7% women, *M*_*age*_ = 31.30, *SD*_*age*_ = 12.91). The majority of these participants (42.2%) had acquired higher education, 46.4% were workers, and 87.3% identified themselves as heterosexual. Additionally, 67% of participants did not know of anyone who had experienced non-consensual dissemination of one’s sexual images, and 97.3% of participants had never been the target of such conduct.

### Procedure and Measures

This study was part of a larger data collection in which other variables were measured for different purposes. Before taking part in the experiment, participants filled out a consent form where they were informed about their right to refuse to participate in the study and to withdraw consent during the study without any consequences. Then, participants were informed that their anonymity would be guaranteed. Once participants agreed to take part in the study, they started filling out the questionnaire. With the exception of the manipulation section, which varied across conditions, all questionnaires had the same headings and questions. Unless otherwise specified, participants stated their level of agreement with each item of the questionnaire on a scale ranging from 1 (= *not at all*) to 7 (= *very much so*).

*Experimental Manipulation*. First, participants were instructed to carefully read a fictitious scenario describing the story of Paola and Giorgio, two 35-year-olds who both worked in administrative positions at a local company where they met. Depending on the experimental condition, Paola and Giorgio had either been in a relationship for five years or had only one date after work. Three years after starting their relationship (vs. during their transient date), Paola asked Giorgio (vs. Giorgio asked Paola) to videotape their sexual activity because she found it exciting, and he reluctantly agreed (vs. he found it exciting and she reluctantly agreed). After some months (vs. after their transient date), Paola decided that she was no longer interested in being in a committed relationship with Giorgio and ended the relationship (vs. telling Giorgio that she was not interested in starting a committed relationship with him). Giorgio was hurt by her decision, as he was deeply in love with her (vs. deeply involved). Ten days after their breakup (vs. a transient date), Giorgio shared the video of their sexual encounter in a work-related WhatsApp chat without Paola’s consent. After this incident, Paola made the decision to report him.

*Manipulation check.* After reading the scenario, we checked the participants’ recall of who asked to record the sexual encounter and whether Paola and Giorgio were in a steady relationship or had a transient date.

*Moral Virtue.* Adopting the procedure used by Pacilli et al. (2017, 2022), we asked participants to what extent they considered Paola trustworthy, honest, sincere, and moral. We calculated a moral virtue index by averaging the responses to the four items (Cronbach’s *α* = 0.89).

*Victim Blaming*. Four items were then used to measure blame attributions toward Paola for what happened (e.g., “Paola is responsible for what happened,” “Paola is to blame for what happened”; Cronbach’s *α* = 0.73; Bernard et al., 2015; Pacilli et al., 2022; Spaccatini et al., 2019; 2023).

*Helping Intentions*. In line with previous studies (Pacilli et al., 2017, 2022; Pagliaro et al., 2016), we measured proclivity to engage in five types of helping behaviors (e.g., “Report what happened to the police,” “Provide Paola with support and assistance”). The global index, created by averaging participants’ answers to the five items after reversing when necessary, was internally consistent (Cronbach’s *α* = 0.64).

*Sexism.* Participants’ sexism was measured with the 12-item version of the Ambivalent Sexism Inventory (ASI; Glick & Fiske, 1996; Rollero et al., 2014). We asked participants to indicate their agreement with each statement (e.g., “Every man ought to have a woman whom he adores"; “Women exaggerate problems they have at work”) on a 6-point scale (0 = *strongly disagree*, 5 = *strongly agree*) (Cronbach’s *α* = 0.88).

*Sociodemographic information.* We finally collected information about participants’ gender, age, sexual orientation, and education.

## Results

We conducted a 2 (Sexual Agency: Low vs. High) × 2 (Relationship’s Length: Steady vs. Transient) × 2 (Participants’ Gender: Man vs. Woman) analysis of covariance (ANCOVA) for each dependent variable, covarying out for participants’ sexism. Means, standard deviation and correlations among the key study variables are presented in Table 1. Differences in the degrees of freedom are due to instances of missing data.

**Table 1:** Means, standard deviations, and correlations between the key variables of the study

	*M*	*SD*	1	2	3	4	5	6	7
1. Relationship’s length^a^		–	–						
2. Sexual agency^b^	–	–	− 0.02	–					
3. Participants’ gender^c^	–	–	0.08*	− 0.07	–				
4. Moral virtue	5.48	1.15	0.12**	− 0.08*	0.16**	–			
5. Victim blaming	2.35	1.23	− 0.20**	0.03	− 0.13**	− 0.43**	–		
6. Helping intentions	5.43	1.13	0.06	− 0.15**	0.17**	0.27**	− 0.26**	–	
7. Sexism	2.08	0.77	− 0.04	0.05	− 0.16**	− 0.29**	0.49**	− 0.09*	–

Moral virtue, victim blaming, and helping intentions scales ranged from 1 to 7. Sexism ranged from 0 to 5

^a^Relationship’s length is coded: 0 = *transient* and 1 = *steady*

^b^Sexual agency is coded: 0 = *low* and 1 = *high*

^c^Participants gender is coded: 0 = *man* and 1 = *woman*

^*^p < 0.05, **p < 0.01

*Moral Virtue.* Contrary to Hp1a, sexual agency did not reach significance, *F*(1, 583) = 1.29, *p* = 0.257, *η*_p_^2^ = 0.002. As hypothesized (Hp1b), results showed a significant main effect of the relationship’s length, *F*(1, 583) = 5.68, *p* = 0.018, *η*_p_^2^ = 0.010. Specifically, when the relationship was transient the moral virtue attributed to the woman described in the scenario was lower (*M* = 5.34, *SD* = 1.17) than when the relationship was steady (*M* = 5.62, *SD* = 1.11). Moreover, we found a main effect of the participants’ gender, *F*(1, 583) = 7.69, *p* = 0.006, *η*_p_^2^ = 0.013. In this regard, when participants were men (*M* = 5.21, *SD* = 1.11) the moral virtue attributed to the woman was lower than when participants were women (*M* = 5.61, *SD* = 1.14). Our findings also show a significant effect of sexism, *F*(1, 583) = 46.76, *p* < 0.001, *η*_p_^2^ = 0.074, indicating that participants with higher sexism attributed less moral virtue to the victim (Beta = -0.41). Furthermore, no two- or three-way interactions between variables were found to be reliable (*Fs* < 1.24, *ps* > 0.265).

*Victim blaming*. In contrast to Hp2a, sexual agency did not emerge as significant, *F*(1, 583) = 0.10, *p* = 0.750, *η*_p_^2^ = 0.000. As expected (Hp2b), the relationship’s length *F*(1, 583) = 18.29, *p* < 0.001, *η*_p_^2^ = 0.030 proved significant. In particular, participants expressed toward the target more blame for what happened when the relationship was transient (*M* = 2.59, *SD* = 1.28) rather than when the relationship was steady (*M* = 2.11, *SD* = 1.13). Sexism emerged as a significant covariate, *F*(1, 583) = 181.644, *p* < 0.001, η_p_^2^ = 0.238, showing that participants with higher sexism attributed more blame to the victim (Beta = 0.78). Neither the main effect of the participants’ gender, *F*(1, 583) = 1.37, *p* = 0.243, *η*_p_^2^ = 0.002, nor the other two- or three-way interactions emerged as significant (*Fs* < 2.57, *ps* > 0.110).

*Helping Intentions*. As expected (respectively Hp3a and Hp3c), a main effect of the sexual agency, *F*(1, 583) = 8.77, *p* = 0.003, η_p_^2^ = 0.015, and the participants’ gender, *F*(1, 583) = 13.02, *p* < 0.001, η_p_^2^ = 0.022, emerged. Participants were less willing to help the woman described in the scenario when the sexual agency was high (*M* = 5.25, *SD* = 1.21) and when they were men (*M* = 5.16, *SD* = 1.24) rather than when the sexual agency was low (*M* = 5.59, *SD* = 1.03) and they were women (*M* = 5.57, *SD* = 1.04). The relationship’s length did not emerge as significant, *F*(1, 583) = 0.50, *p* = 0.480, *η*_p_^2^ = 0.001, as well as the participants’ sexism, *F*(1, 583) = 1.77, *p* = 0.184, *η*_p_^2^ = 0.003. Moreover, no other two- or three-way interactions between the variables considered in the analysis were reliable (*Fs* < 0.34; *ps* > 0.561).

**Fig. 1:**
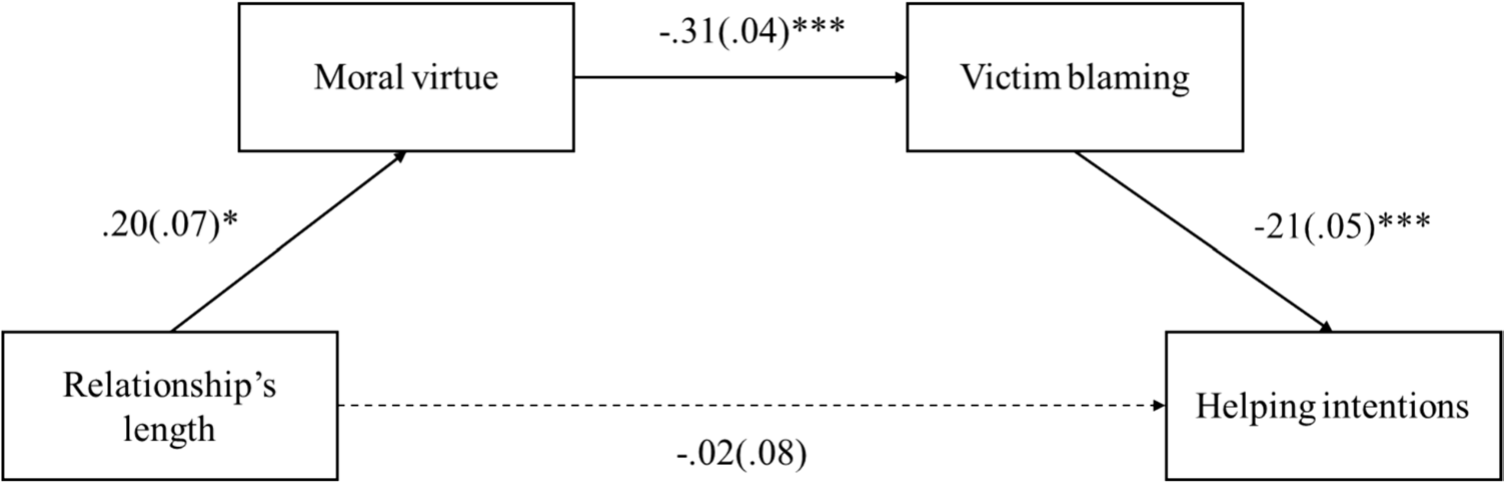
Sequential mediation model illustrating how the indirect influence of relationship length on helping intentions towards the female victim is mediated by attribution of moral virtue and blame towards her. *p < 0.05, ***p < 0.001

*Mediation Analysis*. Finally, we tested a sequential mediational model aimed at analyzing the possible mediating role of moral virtue and victim blaming in explaining the effect of the relationship’s length on helping intentions (see Fig. 1). To this aim, we tested a sequential mediational model using PROCESS Macro (Model 6) for SPSS with 10,000 bootstrapping resamples (Hayes, 2018). Since sexual agency did not affect moral virtue, we decided not to include this variable in the mediation model. Furthermore, given that we used scores with different anchors, we standardized all measures before testing the model. Thus, the relationship’s length (0 = *transient*, 1 = *steady*) was entered as a predictor, moral virtue and victim blaming as sequential mediators, and helping intentions as a dependent variable (Hp4b). Participants’ gender and sexism were considered covariates in the model. Findings indicated that the overall model was significant, *R*^*2*^ = 0.12, *F*(5, 586) = 16.28, *p* < 0.001. The path linking the relationship’s length to moral virtue emerged as significant (*t* = 2.57, *p* = 0.010, 95% CI [0.05 to 0.35]), as did the relationship between this latter variable and the victim blaming (*t* = −8.68, *p* < 0.001, 95% CI [−0.37 to -0.24]). Furthermore, victim blaming influenced helping intentions (95% CI [−0.30 to −11]). The direct effect of relationship’s length on helping intentions did not reach significance (*t* = −0.19, *p* = 0.849, 95% CI [−0.17 to 0.14]), instead, the indirect effect of the relationship’s length on helping intentions through the mediation of moral virtue and victim blaming emerged as significant (95% CI [0.00 to 0.03]). Based on Hayes (2013), we concluded that the lack of a direct effect of relationship length on helping intentions does not compromise the validity of the overall model (Fig. 1).

## Discussion

Individuals who have been subjected to gender-based violence frequently encounter stigmatization in diverse manifestations (Maass et al., 2013; Penone & Spaccatini, 2019; Pina et al., 2009). Accordingly, research has revealed that in cases of sexual assault, there is a tendency to place a disproportionate emphasis on the conduct, motives, and actions of women victims (Abrams et al., 2003; Morabito et al., 2019), with the aim of ascertaining the degree of responsibility they bear for their own victimization. This tendency is even more pronounced for women victims of image-based sexual abuse, as taking or sharing their sexual images may be perceived as acting irresponsibly, promiscuously, and without self-control (Albury & Crawford, 2012; Henry et al., 2021). In the sexuality domain, traditional feminine gender roles indeed have long prescribed that women should exhibit a low sex drive, be sexually passive, and have relatively few sexual needs, which is inconsistent with the traits required for initiating sexual activity, such as assertively communicating one’s desires (Harrington & Maxwell, 2023).

In the present research, we aimed to examine how bystanders perceive a woman victim of image-based sexual abuse according to the extent to which she distanced herself from traditional female sexuality in terms of exhibited sexual agency and the length of her relationship with the man with whom she recorded the sexual intercourse. We focused specifically on the impact of sexual agency and length of relationship on moral evaluation, victim blaming, and willingness to support the victim. Our findings showed that high (vs. low) sexual agency decreased individuals’ helping intentions toward the victim. Differently, the transient (vs. steady) relationship with the perpetrator reduced the woman’s perceived moral virtue and increased the extent to which she was blamed for the incident. Moreover, we found that the duration of the relationship indirectly affected the intention to provide assistance through the mediation of moral virtue and victim blaming. Specifically, the transient (vs. steady) relationship with the perpetrator reduced helping intentions towards the victim through perceived moral virtue and increased blame for the incident.

Thus, consistent with previous research on sexual double standard (Zvi, 2022), we can argue that the less the behavior of victims of image-based sexual abuse conforms to traditional stereotypes of female sexuality, the less likely bystanders will be to recognize the responsibility of the perpetrators, focusing instead on the alleged “immorality” of the victims. Interestingly, while participants’ gender significantly influenced their willingness to help the victim (with women generally showing higher levels of helping intentions than men), it did not affect their levels of victim blaming, thereby highlighting a widespread tendency to conceive women who go beyond traditional sexual activities as somehow responsible in case of violence (Penone & Spaccatini, 2019).

Especially relevant in this context is the fact that in contemporary society, we are witnessing sexual experiences in individuals’ daily lives being increasingly mediated by digital technologies (Spisak, 2023). Despite the increasing prevalence of people who consciously choose to videotape their sexual encounters or engage in practices such as sexting (Hasinoff, 2013), it is often recommended to refrain from engaging in this type of sexual activity in order to avoid potential risks associated with non-consensual dissemination (Ringrose & Regehr, 2023). In this cultural scenario, rather than solely focusing on (legally and culturally) fighting the issue of non-consensual image sharing, an abstinence-based approach has been promoted, which emphasizes the importance of refraining from engaging in the production or distribution of sexually explicit images (Döring, 2014; Krieger, 2017; Setty et al. 2022). As such, because of the stigma associated with engaging in mediated technology-based sexuality and being seen in various states of nude, semi-nude, or sexual situations, victims of image-based sexual abuse are often reluctant to report the crime for fear of shame and social stigma (Campbell et al., 2020; Henry et al., 2021; Ruvalcaba & Eaton, 2020). The need to hide their traumatic experience in order to prevent people’s negative judgment often contributes to promoting victims’ sense of loneliness and social isolation, thus worsening their psychological and physical wellbeing (Bates, 2017; Veronica & Di Giacomo, 2022). In this regard, research has shown that women who experience image-based sexual abuse tend to report significantly lower levels of self-esteem and higher levels of anxiety, post-traumatic stress disorder, depression, and sleep disturbances (Bates, 2017). Given the obstacles women face in reporting image-based sexual abuse, bystanders’ intervention becomes crucial since they play a key role by either assisting the victim or, conversely, doing nothing and condoning the perpetrator’s actions (Banyard, 2011; Burn, 2009; Latané & Darley, 1970; Pagliaro et al., 2020).

Taken together, the present findings underline the need to raise awareness about the deceitfulness of legitimate victims’ images (Carpenter, 2005), targeting people’s levels of ambivalent sexism. Indeed, recognizing stereotypes related to image-based sexual abuse is essential not only at the individual level, for victims who need to receive help, but also at the societal and community level, because only an environment able to identify violence can effectively deal with it (Rollero & De Piccoli, 2020). In order to do so, correct information about gender stereotypes, women’s sexuality, and rape myths should be included in prevention programs and sex education curricula targeted at young adolescents. Moreover, our findings can inform policies targeted at the whole society, creating programs to enhance the knowledge of the sociocultural dimensions underlying the conception of gender-based violence and image-based abuse, thus reducing the stigma and blame faced by victims.

The present research has some limitations that need to be considered in order to develop further studies on the phenomenon. First, image-based abuse may have peculiar dimensions compared to other forms of gender violence that were not examined comparatively in our study and deserve further attention. Future studies should delve deeper into whether victims of image-based sexual abuse are more susceptible to blame and negative moral evaluations compared to other types of sexual abuse victims. Second, in our paper, we only considered violence within heterosexual relationships. Future research should also consider sexual violence within same-sex couples. Indeed, while similarities between heterosexual and lesbian, gay, and bisexual violence were found, unique features and dynamics are present in lesbian, gay, and bisexual relationships, even in regard to image-based abuse (Rollè et al., 2018). Third, future ad hoc research might also be designed to investigate whether and how ideological beliefs moderate the effects we reported, considering the bulk of research showing that conservatives are more likely to be lenient toward perpetrators of gender-based violence due to their endorsement of a traditional masculine worldview (Rollero et al., 2021). Despite these limitations, our findings highlight the importance of looking into how the behavior of female victims of image-based sexual abuse may affect negative attitudes toward them. Furthermore, our study highlights the need to recognize that the perception of women as immoral when they deviate from traditional sexual norms serves to symbolically punish their sexual agency. We must acknowledge and confront the subtle yet insidious hierarchy of acceptable and unacceptable sexual conduct, as it reinforces traditional gender norms (Hasinoff, 2013) and implicitly conveys a powerful message that women’s sexual freedom should be constrained.

## Data Availability

The dataset is available here: https://osf.io/h25w7/files/osfstorage?view_only=1030427729ee4453a13f798c8dae86eb

## Code Availability

SPSS22 was adopted to analyze the data.
